# Camera traps and GPS telemetry can provide similar estimates of daily travel distance in terrestrial mammals

**DOI:** 10.1186/s40462-026-00690-6

**Published:** 2026-07-31

**Authors:** Thomas Osinga, Henrik J. de Knegt, Francesca Cagnacci, Massimo Scandura, Maik Henrich, Marco Heurich, Astrid Olejarz, J. Marcus Rowcliffe, Patrick A. Jansen

**Affiliations:** 1 Department of Biology, Utrecht University, Padualaan 8, Utrecht, 3584 CH the Netherlands; 2 Department of Environmental Sciences, Wageningen University & Research, Wageningen, The Netherlands; 3 Animal Ecology Unit, Research and Innovation Centre, Fondazione Edmund Mach, San Michele all ‘Adige, Italy; 4 NBFC National Biodiversity Future Centre, Palermo, Italy; 5 Department of Veterinary Medicine, University of Sassari, 07100 Sassari, Italy; 6 Department of National Park Monitoring and Animal Management, Bavarian Forest National Park, Grafenau, Germany; 7 Chair of Wildlife Ecology and Wildlife Management, University of Freiburg, Freiburg, Germany; 8 Institute for Forest and Wildlife Management, Inland Norway University of Applied Sciences, Koppang, NO-34 Norway; 9 Department of Game Management and Wildlife Biology, Faculty of Forestry and Wood Sciences, Czech University of Life Sciences, Kamýcká 129 Prague 6, Suchdol, 165 00 Czech Republic; 10 ZSL Institute of Zoology, Regent’s Park, London, NW1 4RY UK; 11 Deptartment of Genetics, Evolution and Environment, University College London, Gower Street, London, WC1E 6BT UK; 12 Smithsonian Tropical Research Institute, Luis Clement Avenue 401 Tupper building, Ancon, Panama

**Keywords:** Ctmm, Diel activity, Movement speed, Path tortuosity, Day range, Camera trap, Gps telemetry, Daily distance travelled, Eulerian, Lagrangian

## Abstract

**Background:**

Two primary methods exist for estimating the daily travel distance by free-ranging animals: inference from animal location data recorded with biologging or wearable GNSS tags (e.g. GPS), and inference from snapshots of animal movement recorded by stationary sensors (e.g. camera traps). Formal comparisons between the two approaches, which differ in both their assumptions and in choices made during analyses, remain scarce.

**Methods:**

Here, we compare GPS- and camera-based estimates of daily travel distance at matched temporal scales under both simulated and real-world scenarios. First, using simulated trajectories, we varied the GPS sampling interval and estimation method — continuous-time movement models (CTMM) vs. straight-line displacement (SLD) — as well as camera-analysis choices (activity correction, data sparsity) to test how well each approach recovers the true distance travelled. Second, we applied the same workflow to empirical datasets with overlapping GPS telemetry and camera-trap observations from the same populations.

**Results:**

We found that, under the simulated movement and observation scenarios considered here, daily distance travelled could be estimated with limited bias from both camera-trap and GPS data using CTMM, but not with GPS data using SLD. Accurate estimation required correction for GPS-error, sufficiently high GPS sampling frequency, activity estimation for camera-trap data and adequate camera-trap data volume to estimate instantaneous speed and activity. Using empirical data from three case studies, we found that camera trapping and GPS-based CTMM estimates were of similar magnitude with overlapping confidence intervals, while GPS-based SLD estimates were lower than the others.

**Conclusions:**

These findings suggest that, when appropriately implemented, both methods can yield similar and fairly unbiased estimates of daily travel distance. These results provide practical guidance for using and potentially combining GPS-based CTMM and camera trapping-based travel distances in density models such as REM (random encounter model), strengthening ecological inference and the effectiveness of wildlife monitoring and management.

**Supplementary Information:**

The online version contains supplementary material available at https://doi.org/10.1186/s40462-026-00690-6.

## Background

Movement is a fundamental aspect of animal ecology, linking space use, behaviour, and species interactions [1]. Animal movement can be described using fine-scale metrics, including step length, turning angle, velocity, and speed, typically measured at sub-minute to hourly intervals, each capturing different properties of the underlying movement process. These metrics play a central role in ecological inference because they reflect how animals allocate time and energy, respond to environmental conditions, and interact with their environment. In particular, speed-based movement metrics are widely used to infer behaviour, energetics, and habitat use [2, 3], to reveal responses to resource distribution, human disturbance or natural predators [4, 5], and to inform energetic models by quantifying trade-offs between resource acquisition and energy expenditure [6]. They are also increasingly used to evaluate how landscape structure and fragmentation shape animal movement and ecological connectivity [7].

While these fine-scale metrics provide insight into short-term movement processes, movement can also be summarized over broader temporal scales to capture longer-term outcomes. One commonly used summary metric is the daily distance travelled or daily travel distance (DTD), the total distance travelled over a 24-hour period [8]. DTD is a key parameter in population density models such as the Random Encounter Model (REM; [9–11]) and has also been used in comparative studies of animal energetics and space use [2]. As a time-integrated metric, DTD reflects the cumulative expression of fine-scale movement, regardless of whether that movement is characterized through step length, speed, or velocity-based representations of the movement process. Despite its widespread use, DTD cannot be observed directly and must be inferred from movement data collected at discrete temporal intervals. Consequently, understanding the performance and limitations of movement-derived estimators is an important component of validating DTD and other movement metrics (e.g. Whetten et al., [12].

Two primary methods exist for estimating DTD in free-ranging animals. The first is a Lagrangian approach, which estimates DTD from a trajectory consisting of successive locations obtained by tracking individuals with biologging, most commonly, GPS telemetry devices. The second is an Eulerian approach, in which distance travelled is inferred from image sequences recorded with camera traps [11, 13–15]. These methods have very different strengths and weaknesses.

DTD estimation derived from GPS-based trajectories typically involves calculating the straight-line displacement (SLD) between successive fixes divided by the time elapsed [16]. While simple, this approach has well-known drawbacks. At coarse sampling intervals and/or when animals move in tortuous paths, SLD underestimates the true distance travelled between fixes [11, 15, 17]. Increasing the sampling frequency reduces this bias, but very high frequencies (i.e., very short intervals) amplify the influence of GPS error, which can artificially inflate DTD estimates [18, 19]. Moreover, SLD-based estimates are typically arithmetic values, without confidence intervals for observation bias or uncertainty, complicating comparisons across individuals, or studies. These limitations stem from the fact that the observation model implied by SLD is a poor match to the data-generating process; animal movement is continuous and autocorrelated [3, 20, 21], yet we observe it only as a discrete sequence of locations with error. Consequently, the path reconstructed by connecting successive fixes omits fine-scale structure in the trajectory. A true trajectory reflects complex responses to stimuli and, in cognitively advanced species, navigation decisions [1]. Direction, tortuosity, acceleration and decelerations are components of the movement trajectory that emerge as a sequence of instantaneous speed vectors [22], but much of this information is lost when movements are reduced to straight lines between fixes [18]. This makes the estimation of movement parameters non-trivial when derived directly from empirical tracks.

To overcome these challenges, continuous-time movement models (CTMM; [23]) have been developed. By separating the underlying movement process from the discrete sampling schedule, CTMM can provide scale-insensitive (i.e., estimates not dependent on fix rate) and unbiased estimates of DTD, provided that the fix interval is sufficiently short relative to the characteristic autocorrelation timescales of movement and that GPS error is properly accounted for [18, 23]. This robustness arises because CTMM fits a latent, autocorrelated movement (velocity) process to the observed, discretely sampled locations, rather than treating straight-line segments between fixes as the animal’s true path. However, this scale-insensitivity is not guaranteed under all conditions. When sampling intervals become very coarse relative to the rate of change of velocity, the model lacks information on short-lag behaviour, therefore estimation accuracy is expected to degrade (e.g. see female roe deer in the analysis of [24]). Thus, although CTMM is far more robust than SLD to variation in sampling rate, its performance may still be constrained by the temporal resolution of the input data.

The second method for estimating DTD, the Eulerian approach with camera traps, relies on photogrammetry to convert the movement of animals passing through the camera view from pixels to real-world distances [25], then divides this by the time between frames to obtain speed (e.g. using the protocol in [26]). Because the time between frames can typically be set to be very small (e.g. <1 s; [14]), camera-trap data can get mean speed estimates at a temporal resolution high enough to better approximate the underlying continuous movement trajectory [11, 18, 21]. However, because cameras almost always capture active animals, speed observations measured in this way provide an estimate of average speed while active. DTD can then be derived by multiplying the speed while active by estimates of daily activity (the proportion of time spent active per day), which can be obtained directly from the temporal distribution of camera-trap records [27].

The camera-trap approach is an attractive alternative to biologging because it is less invasive (e.g. no need to capture animals), more cost-effective, capable of covering large spatial scales, can gain population-wide estimates, and cover the full suite of large and middle-sized terrestrial mammals. However, estimates of camera-trap analysis are sensitive to factors such as camera placement, detection probabilities [28], and the temporal resolution of image sequences [29], as well as behavioural reaction to the presence of the camera. In addition, uncertainty from photogrammetric calibration and user annotation is not quantified in existing camera-trap speed analyses, as no general method for doing so currently exists (but see Rowcliffe et al., in prep). Finally, collecting sufficient data can be difficult in practice, especially for some species occurring at low density [30]. Thus, while promising, this approach remains less standardised than GPS-based methods.

The two approaches differ in their assumptions, sampling resolutions, and potential biases, and there is no established gold standard for validation. Both frameworks have been tested independently, typically under simulation settings aligned with the assumptions of the respective estimators [11, 18, 21], yet comparatively little is known about how they perform when evaluated against movement processes not generated directly from the assumptions of either estimator. Such conditions are likely more representative of empirical ecological data. Formal comparisons between DTD estimates derived from camera-trap and GPS telemetry data remain scarce (but see [11, 15]). Evaluating both approaches under matched spatial and temporal conditions provides an opportunity to determine if they can produce similar estimates of DTD, identify the circumstances under which they diverge, and assess the practical consequences of methodological choice. Understanding these relationships is essential for determining the conditions under which each approach provides reliable DTD estimates, and for guiding when they can be used as alternatives or complementary sources of information.

In this study, we aim to understand the ability of Lagrangian (GPS-based) and Eulerian (camera trap-based) approaches to estimate DTD at matched temporal scales under both simulated and real-world scenarios. Our study is structured around three questions. First, using simulated (thus known) trajectories, we test the accuracy of both Lagrangian and Eulerian approaches under idealized conditions (i.e., a high-frequency 5-minute interval for telemetry data estimated using both CTMM and SLD; and perfect knowledge of activity for camera trap data). Second, we identify the mechanisms of bias and uncertainty by varying data properties (e.g. positional error, fix rate and number of fixes, sparse camera detections, and activity estimation) to determine how and why estimation accuracy and precision deteriorate. Third, we apply the same workflow to empirical datasets from two species with overlapping GPS and camera-trap observations to evaluate whether the two approaches yield similar DTD estimates when applied to real populations. The results provide practical guidance on the conditions under which each approach produces reliable DTD estimates for use in density models such as REM, thereby strengthening ecological inference and supporting wildlife monitoring and management.

## Methods

### Simulation study

**Fig. 1:**
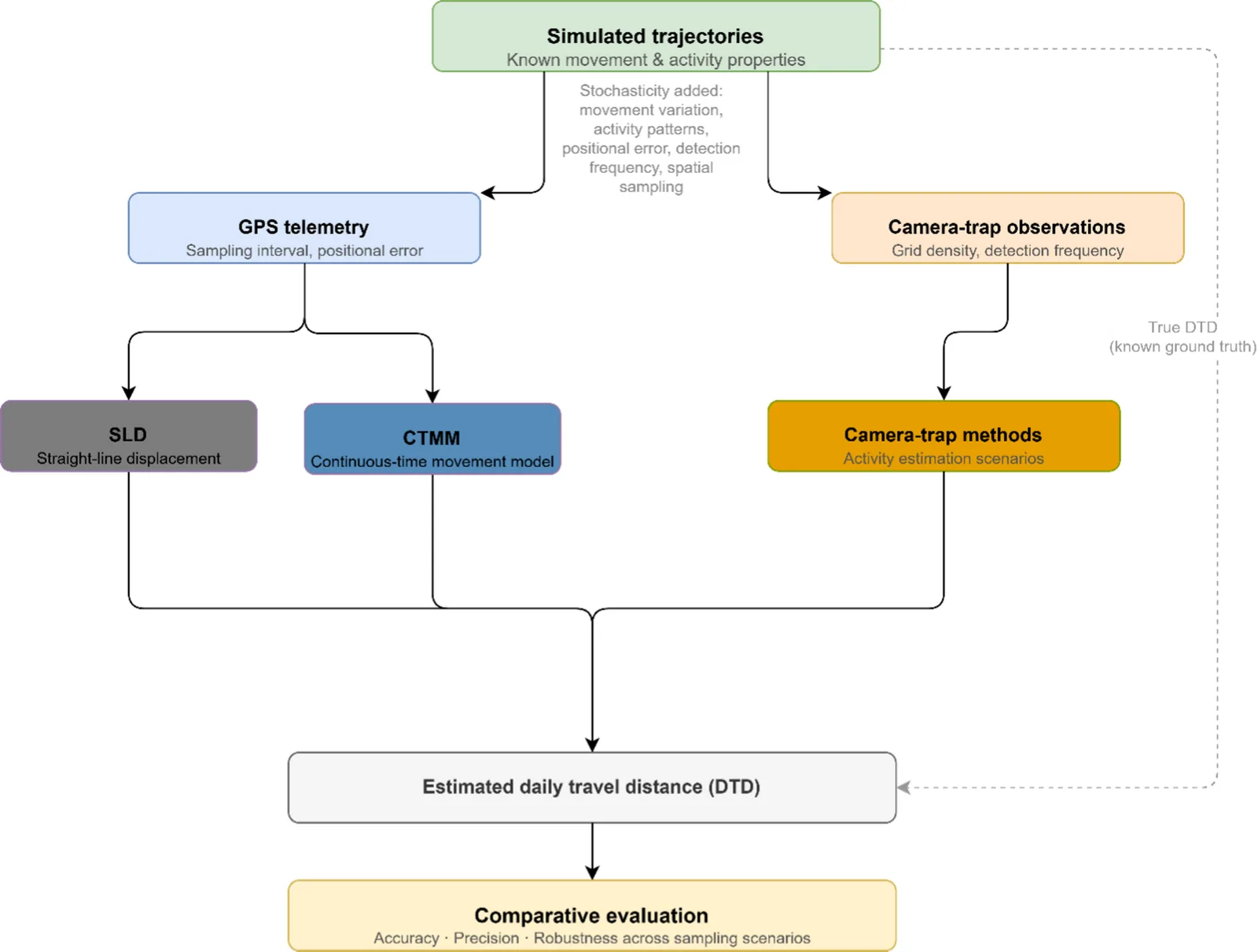
Overview of the simulation study framework, in which simulated animal trajectories served as shared ground truth for both observation types. GPS-derived estimates (SLD and CTMM) and camera-trap-derived estimates were each compared independently against the true DTD, allowing direct evaluation of method accuracy and cross-method comparability under varying sampling conditions

The simulation study was designed to evaluate how accurately and comparably GPS- and camera-trap-based approaches estimate DTD under different observation conditions (Fig. 1; Table 1). Rather than constructing simulations directly from the assumptions of either estimation framework, we developed a generative movement model intended to approximate continuous animal movement. The modelling approach was designed to preserve the key properties both frameworks are designed to estimate: speed, activity patterns, and temporal autocorrelation. The resulting data-generating process was therefore not fully captured by either method, but retained sufficient behavioural and observational structure for both frameworks to operate meaningfully. GPS fixes and camera-trap observations were then sampled from the same underlying trajectories (similar to [31]), allowing both estimation approaches to be evaluated against a shared set of known movement paths rather than against simulations constructed around the assumptions of each framework separately. The simulations additionally incorporated behavioural heterogeneity, and variable sampling conditions characteristic of empirical movement data.

### Simulation of animal movement and camera trapping data

To compare GPS- and camera-trap- based DTD estimation, we simulated a 20 km x 20 km landscape with moving animals, whose positions were sampled by simulating a measurement process with wearable GPS devices as well as stationary camera traps. We ran 200 replicates to characterize robustness of model outputs.

**Table 1:** Overview of simulation scenarios used to evaluate daily travel distance (DTD) estimation from GPS telemetry and camera-trap data

Analysis	Sub-analysis	Levels	Replicates
SLD performance	GPS fix interval	1, 2, 3, 4, 5, 15, 30, 60, 120, and 240 min	200
CTMM performance	GPS fix interval	5, 15, 30, 60, 120, and 240 min	200
	Positional error	Error-free; simulated GPS error – (Supplementary information S2; Fig. S3)	25
	GPS-tag duration	30, 50, 70 days – (Supplementary information S2, Fig S2)	25
Camera-trap performance	Camera density	2 × 2, 4 × 4, … 24 × 24 camera grids spanning the study area	200
	Activity treatment	No correction; estimated activity; true activity	200
	Amplitude varied	0, 0.2, 0.4 – (Supplementary information S2; Fig. S1)	25

In each replicate, we simulated populations of 50 individuals by drawing individual home-range centres across the simulated area using complete spatial randomness. Individual animal trajectories were simulated at a 1-s resolution for 50 days using an Ornstein–Uhlenbeck Foraging (OUF) process [21] with a circadian modulation of movement speed. The OUF process parameters included a positional autocorrelation timescale ($\:{\tau\:}_{pos}$ = 6 h), a velocity autocorrelation timescale ($\:{\tau\:}_{vel}$ = 0.01 h) converted to seconds. To impose circadian variation in speed, we applied a baseline speed distribution defined by a log-gamma envelope (shape = 2, scale = 0.1) plus a sinusoidal amplitude (amp = 0.2) varied according to a sinusoidal function with amplitude 0.2 over a 24 h period (similar to [32, 33]). Each day included a rest window during which animals were stationary, with the start and end times randomly drawn from 07:00–10:00 and 14:00–16:00, respectively, yielding an average active period of ~ 69% of the day. A “homing” force toward each individual’s starting location (strength = $\:\frac{dt}{{\tau\:}_{pos}}$), provided home-range bias, with a small gaussian perturbation (mean 0, SD 0.01) added to the drift term to introduce variability in the directional pull. These additions to the movement model alter the autocorrelation structure of the simulated trajectories, but serve to produce circadian and rest-driven variation in movement and activity relevant for daily travel distance estimation. Displacements and true speeds were recorded at each step, resulting in home-ranging foragers with a true average daily travel distance (DTD) of approximately 16 km.

To obtain camera data, we simulated 12 regular camera grids ranging from 2 × 2 to 24 × 24 cameras covering the same area. A camera recorded a detection whenever the simulated path entered a 15 m radius around the camera. Detections were grouped into sequences per camera using a 2-minute rule: consecutive detections separated by ≤ 2 min were assigned to the same sequence, while a gap > 2 min initiated a new sequence. For speed and activity estimation, we retained only sequences containing at least two detection within 15 m of the camera. Sequence-level speeds were then obtained by dividing travel distance within the detection area by duration, resulting in a (near instantaneous) speed while active in the detection area. We did not simulate any error in the detection process or in the inferred location of animals for each trigger within a sequence, such that camera-trap observations reflected idealized conditions.

To obtain lower-frequency GPS results, we down-sampled each simulated 1-s resolution track to one fix per 1, 2, 3, 4, 5, 15, and 30 min, and 1, 2 and 4 h. At each retained fix, a Dilution of Precision (DOP) value was drawn from a log-normal distribution (meanlog = 0.5, sdlog = 1) and converted to positional error (σ = 3 × DOP) (metres). Independent Gaussian noise with SD σ was added to the true position; fixes with DOP > 10 were discarded (~ 1.6% of fixes).

### Analysis of simulation data: cameras

DTD was calculated from camera-trap data following the approach in Rowcliffe et al., [10], which involves combining diel activity and near instantaneous movement speed. Diel activity patterns from simulated camera trapping data were estimated using circular kernel density methods (activity package; [27, 34]). Speed was obtained from simulated camera-trap data using the methods described by Rowcliffe et al., [11]. To obtain the mean speed (m s⁻¹) and its SE, we fitted a size-biased log-normal distribution by maximum likelihood (bbmle v.1.0.25.1, [35]) to the sequence-mean speeds, which accounts for the fact that faster movements are more likely to produce a detection. Finally, we combined activity and speed to estimate the mean DTD:

$$\:DTD\:\left(km\right)\hspace{0.25em}\:=\hspace{0.25em}\:v*a*\frac{86400\:s\:}{1000\:m\:k{m}^{-1}}$$

where * v* is mean speed in $\:m\:{s}^{-1}$, * a* is the estimated active proportion of the day, and $\:\frac{86400\:s}{1000\:m\:k{m}^{-1}}\:$converts $\:m\:{s}^{-1}$ to * km* [10, 11].

We calculated three versions of DTD: one without activity correction, one corrected using activity estimated from camera detections, and one using the true activity. The standard error of DTD when using the estimated activity was computed using the delta method [36], which propagates uncertainty from both the speed and activity estimates. Variance from the speed model was combined with the standard error of the activity estimate, assuming independence between the two components. This approach provides an efficient first-order approximation to DTD uncertainty, avoiding the considerable computational burden associated with repeated joint bootstrap resampling. To evaluate the validity of this approach, we simulated 400 DTD estimates across three simulation scenarios and compared delta-method standard errors with joint bootstrap estimates obtained by resampling both components simultaneously. Delta-method estimates closely matched bootstrap-derived standard errors across scenarios, with only slight underestimation at low sample sizes (< 100 sequences; Supplementary information S2; Fig S4). Overall agreement was high, with bootstrap estimates deviating only marginally from the 1:1 expectation (mean deviation ≈ 1.04). For the two alternative DTD estimates that did not use estimated activity (i.e., DTD calculated without activity correction and DTD calculated using true activity), standard errors were derived solely from uncertainty in the speed estimates.

### Analysis of simulation data: GPS telemetry

The SLD-based DTD (km) estimate was calculated for each subsampled dataset of each of the 50 simulated individuals by summing the distances between consecutive fixes and dividing by day length, and summarized across individuals to obtain the population mean and standard error.

The CTMM-estimate was calculated for eight randomly selected individuals, using the ctmm R package ([23]; version 1.3.0). For each individual, we used five of the subsampled trajectories (5, 15, 60, 120, and 240 min). GPS error variance was estimated from simulation periods when animals were always stationary (10:00–14:00), enabling separation of observational error from true movement variance. We fitted a candidate set of continuous-time movement models to each subsampled track using the workflow described by Fleming et al., [21]. The candidate set includes the independent identically distributed (IID) model, Brownian motion (BM), Ornstein–Uhlenbeck range-resident process (OU), integrated Ornstein–Uhlenbeck velocity process (IOU), and the Ornstein–Uhlenbeck Foraging model (OUF). Model selection was based on the lowest AICc, with IOU and OUF preferred when support was comparable, as these explicitly incorporate velocity autocorrelation.

Estimates of DTD were only derived from models with a velocity term (IOU, OUF). Simpler models (IID, BM, OU) were still included in the candidate set because they serve as diagnostic indicators of data quality: if a simpler model is selected over IOU or OUF, it implies that the sampling interval is too coarse relative to the animal’s autocorrelation timescales to resolve velocity structure [18]. In other words, the data do not contain sufficient short-lag information to reliably estimate velocity autocorrelation. Simpler movement models such as IID, Brownian motion (BM), or Ornstein–Uhlenbeck (OU) processes describe movement solely in terms of positions, without an explicit velocity process. While DTD can be estimated under such models, the absence of a velocity process prevents recovery of fine-scale movement tortuosity between observation times, leading to systematic underestimation of time-integrated speed and hence DTD. When these models were selected, the track was therefore deemed insufficiently resolved to support unbiased DTD estimation, and the corresponding individual was excluded from further analysis.

Individual-level DTD estimates were then synthesized using the meta-analysis functionality in ctmm [23, 37], which combines estimates across individuals while propagating estimation uncertainty, yielding population-level mean DTD and confidence intervals. This framework allowed direct comparison of DTD estimates from straight-line displacement (SLD), continuous-time movement models (CTMM), and camera trapping against the true simulated values.

### Comparison of simulation estimates

To evaluate and compare the performance of each estimation approach, we computed a set of summary statistics across simulation replicates. Bias was calculated as the mean difference between estimated and true DTD (mean estimate − mean truth), and relative bias as bias expressed as a percentage of the true DTD. Precision was quantified as the standard deviation of estimates across replicates. Root mean square error (RMSE) was calculated as combined measure of accuracy and precision. We additionally reported mean CI width and coverage, defined as the proportion of replicates in which the true DTD fell within the estimated 95% confidence interval.

### Empirical test

#### Study sites and data

We applied our speed estimation workflow to camera-trap and GPS data from two temperate forest sites: Bohemian Switzerland National Park (BSNP) in Czech Republic, and Bavarian Forest National Park (BFNP) in Germany. At both sites, camera traps recorded red deer (*Cervus elaphus*) at the same time as GPS data was available for the species, and in BFNP this was also the case for wild boar (*Sus scrofa*). Camera traps were active during summer 2024 at both sites. At BFNP, 60 cameras were deployed and data from 1 June to 25 September 2024 were used in this analysis, representing a subset of the full deployment period (Supplementary Information S1). At BSNP, 33 cameras were deployed from 10 June to 13 August 2024; cameras were relocated after four weeks, resulting in 66 unique deployment locations sampled over the full eight-week period. GPS collars recorded animal locations at sampling intervals ranging from 5 min to 2 h. At BSNP, GPS data were available for 10 red deer, while at BFNP data were available for 15 red deer and 7 wild boars. While this sample size likely represents a small proportion of the local population and is unlikely to be perfectly representative, it introduced a comparable inter-individual variability as the simulated case. The camera trap survey design followed the EOW-protocol [38]. A full technical description of the GPS telemetry and camera-trap methodologies, including site-specific implementation details, is provided in Supplementary information S1.

Camera-trap data were processed following the same workflow as in the simulation study (see above), with detections grouped into sequences separated by gaps > 2 min and speeds modelled under a size‐biased lognormal distribution [11]. GPS data were analysed with the same two approaches as in the simulation study. As before, CTMM estimates were used when a velocity term was supported, and SLD provided interval-based average DTD. Model settings followed the simulation analyses, except that location error variance (σ) was estimated directly from GPS collar DOP values using an initial guess error of 10, following the recommended value for GPS data [39].

Spatial and temporal overlap between GPS-tracked individuals and the camera trap array was ensured by retaining only fixes falling within the geographic extent of the camera trap deployment locations plus a ~ 2.5 km buffer, and within the period bounded by the earliest and latest camera trap deployment dates at each site. Only individuals retaining more than 1,000 GPS fixes after applying these filters were included in the analysis (Supplementary information S1; Figure S1). This resulted in 5 wild boar and 14 deer in BFNP, and 10 deer in BSNP.

Full workflow details, model settings, and simulation outputs are provided in our online repository [40].

## Results

### Performance under idealized simulated conditions

**Fig. 2:**
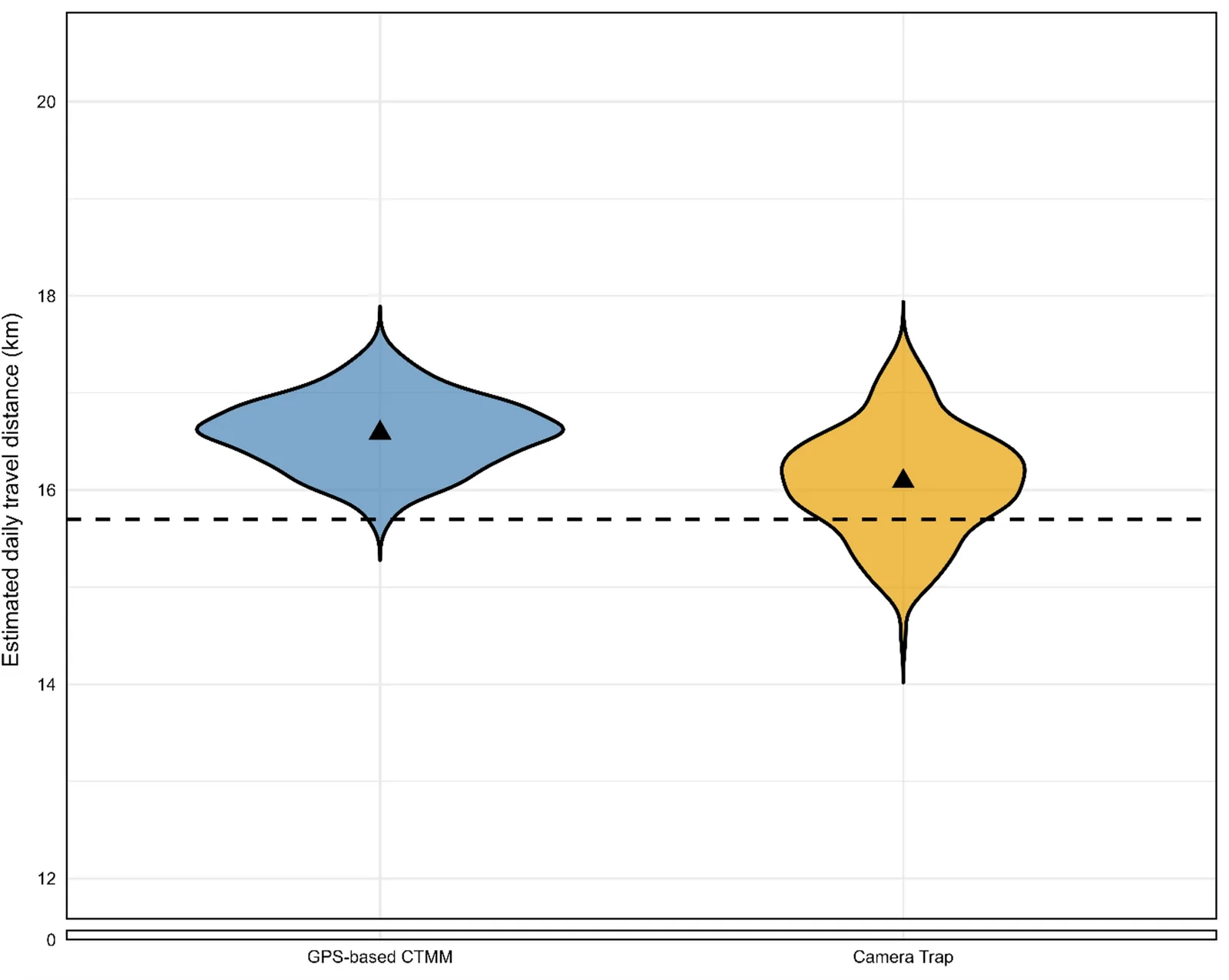
Estimated daily travel distance (DTD) across 200 simulated animal movement scenarios. Violin plots show the distribution of simulation-level estimates, with points representing mean estimated DTD. The black dashed line marks the mean true DTD across simulations. “GPS-based CTMM” estimates were derived from 5-min subsampled trajectories with measurement error correction and based on 10 individual trajectories per simulation, whereas “Camera Trap” estimates used the true activity proportion and approximately 1500 movement sequences per simulation for speed estimation. The y-axis is truncated to improve visualisation of the limited differences among estimators

Camera-trap–based estimates and GPS-based CTMM both recovered DTD values close to the simulated truth under the idealized conditions, with slight positive bias (2.75% and 5.88%, respectively; Supplementary files S2: Table S1, Fig. 2).

### Sensitivity to sampling design and data properties

**Fig. 3:**
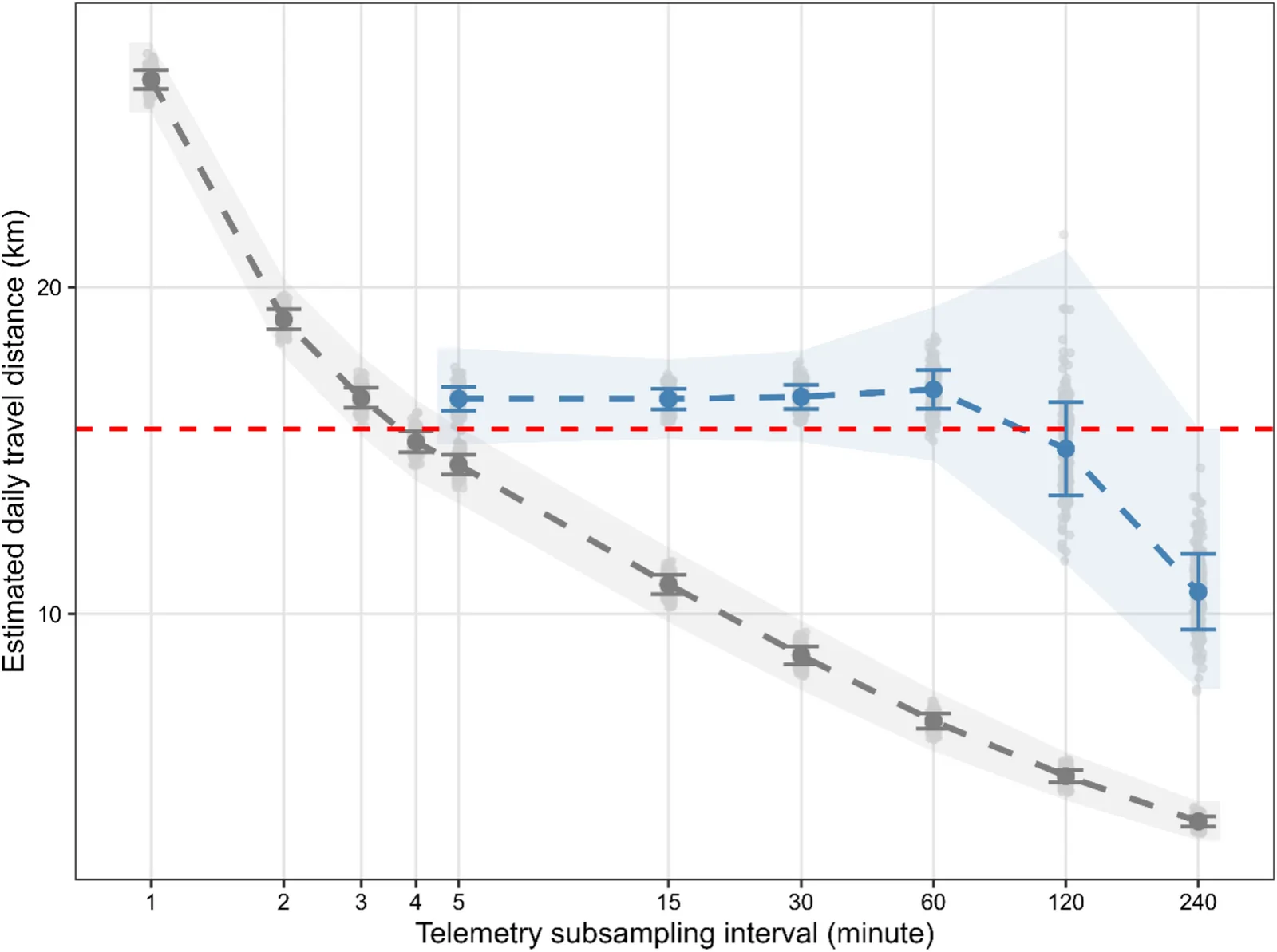
Estimated daily travel distance (DTD) as a function of telemetry subsampling interval across 200 simulated movement scenarios. Grey points and the grey dashed line represent GPS-based estimates derived from straight-line displacement (SLD), whereas blue points and dashed line represent CTMM-based estimates with measurement error correction. The red dashed horizontal line indicates the mean true DTD across simulations. Points show mean estimated DTD across simulations, error bars represent 95% percentile intervals around the across-simulation mean, and faint background points show individual simulation estimates. Shaded ribbons represent the range encompassing 95% of confidence intervals derived from the individual simulation estimates

**Fig. 4:**
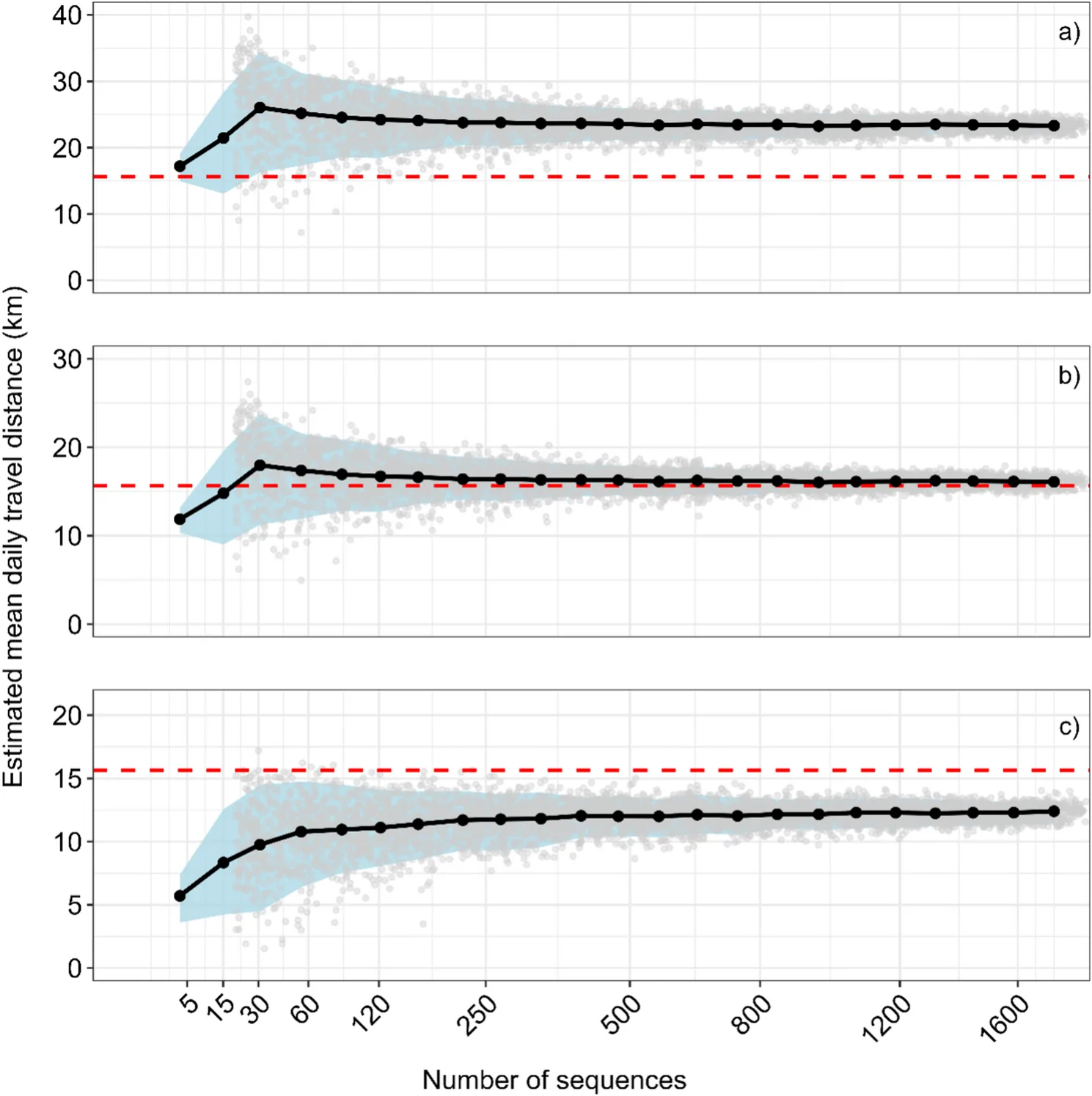
Estimates of daily travel distance (DTD; y-axis) from 200 simulated camera-trap datasets as a function of the number of sequences (x-axis; square-root–transformed) used for speed and activity estimation under three activity correction scenarios: (**a**) no correction, (**b**) true activity information, and (**c**) estimated activity. The red dashed line indicates the true DTD, the black line shows the mean across simulations, the black points indicate the mean of the simulation scenario (camera arrays from 2 × 2 till 24 × 24), grey points represent individual simulation means, and the blue shaded region denotes the 5th–95th percentile range of estimates across simulations

The simulations revealed clear differences in estimated DTD between methods when sampling effort varied (i.e. the number of cameras and GPS fix rate; Figs. 3 and 4). GPS-based SLD estimates were highly sensitive to sampling interval. At short intervals (≤ 2 min), SLD overestimated DTD due to GPS errors, resulting in inflated small-step lengths. At longer intervals (≥ 5 min), it progressively underestimated movement missing tortuous paths, with estimates falling to less than half of the true value at ≥ 120 min. GPS-based CTMM estimates, in contrast, provided more stable estimates across intervals. Mean estimates were generally close to the simulated value (bias < 10% [about 1,5 km]; Supplementary files S2: Table S2) for sampling intervals up to 60 min, with overlapping confidence intervals. At coarser intervals (120–240 min), CTMM estimates declined and uncertainty increased, though the bias remained much smaller than for SLD. Taken together, the simulation indicates that CTMM approaches are robust to variation in GPS sampling schedules, whereas SLD estimates are strongly scale-dependent.

Because DTD estimates were only taken forward when model selection supported a velocity-containing movement model, the number of simulated tracks contributing to the analysis varied among fix intervals. Retention of simulated tracks was near-perfect at fix intervals up to 30 min, where all 10 tracks were retained in every simulation run. At 60 min, mean retention remained high (mean = 9.80 per simulation, minimum = 8), but declined notably at coarser intervals (120 min: mean = 8.22, minimum = 5; 240 min: mean = 8.09, minimum = 5), reflecting the increasing likelihood that velocity-containing models were not supported when temporal resolution was insufficient. Patterns at fix intervals of 60 min and finer are therefore considered robust, while results at 120 and 240 min should be interpreted in light of the reduced and more variable track retention.

Camera-trap–based estimates were highly sensitive to both sampling effort and accuracy of the activity-level estimate. Any bias in activity estimation propagated directly into biased day-range estimates. In our simulations, activity was systematically underestimated because movement speed followed a cyclic pattern over the day (Supplementary information S2; Fig. S1). Only when activity was fixed at its true value and sampling effort exceeded 100 independent speed estimates, DTD estimates were unbiased and showed narrow percentile intervals (14–18 km; Fig. 4).

### Consistency between methods in empirical data

**Fig. 5:**
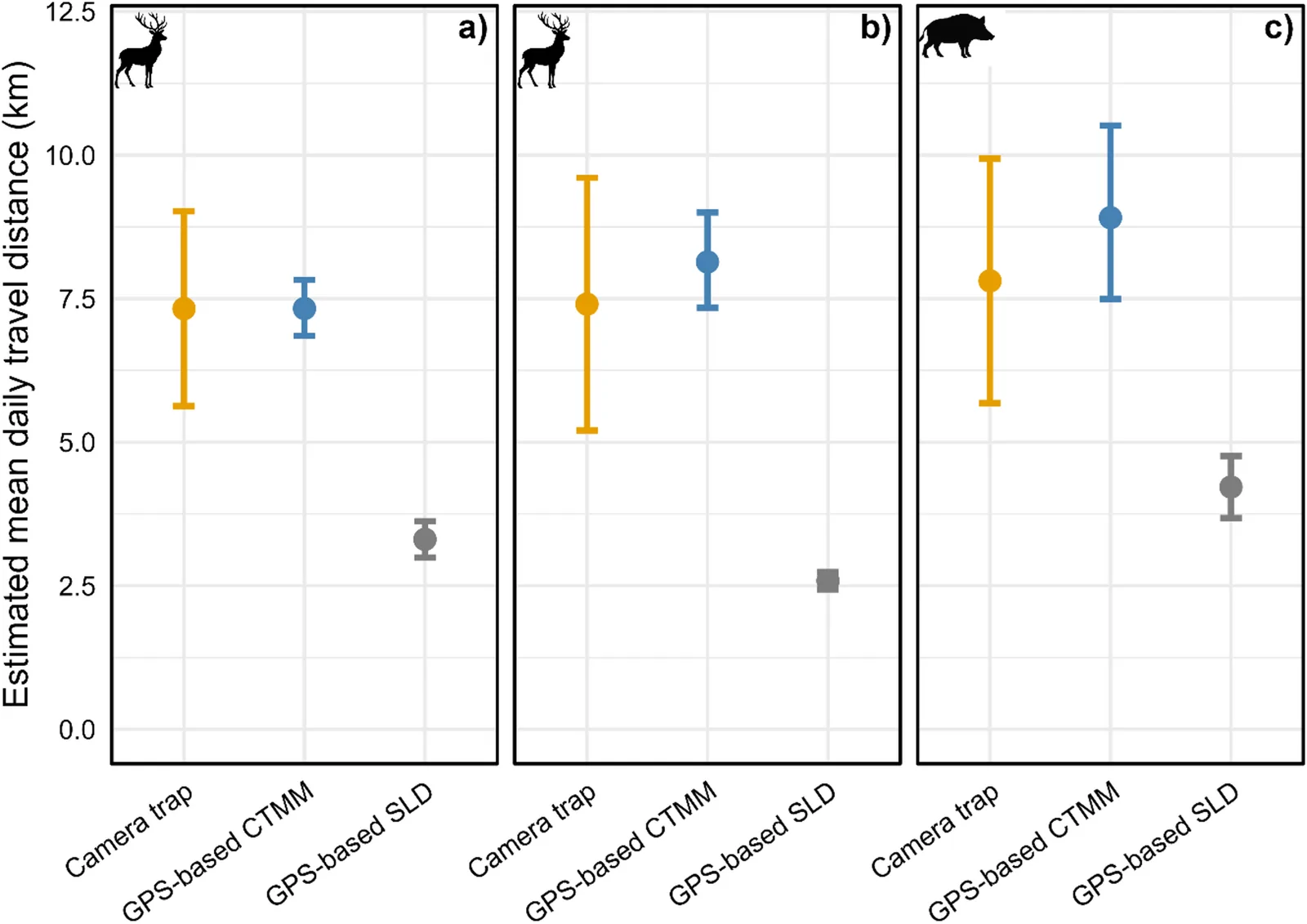
Estimated daily travel distance derived from empirical camera-trap data (y-axis) plotted against GPS-derived estimates (x-axis) for: (**a**) red deer in Bohemian Switzerland, (**b**) red deer in Bavaria, and (**c**) wild boar in Bavaria. Whiskers represent 95% confidence intervals. The number of sequences used for the speed and activity analyses for the camera trap estimates, respectively, were: (**a**) 385 and 1200, (**b**) 154 and 608, and (**c**) 95 and 590. The number of CTMM tracks included in the analyses were: (**a**) 10, (**b**) 14, and (**c**) 4. GPS fix intervals ranged from 5 min to 2 h. For wild boar, fixes were collected every 15 min, whereas for red deer the interval was typically 1 h, with occasional high-frequency sampling windows of 5 min between fixes once per week

DTD estimates from camera traps and GPS telemetry showed broadly consistent patterns across all sites and species (Fig. 5; Table 2). Camera-trap–based and GPS-based CTMM estimates were of similar magnitude, while GPS-based SLD consistently produced lower DTDs, consistent with the bias observed in our simulation results.

Camera traps produced wider confidence intervals (on average ~ 40% of the mean), reflecting that the sites had far fewer usable speed estimates from cameras than from GPS telemetry, and the additional uncertainty from activity correction. GPS-based CTMM estimates were more precise, but these were based on a limited number of collared individuals, of which almost all were female. Despite these differences in certainty, the mean values from both approaches generally agreed well across case studies (Fig. 5; Table 2).

Overall, the empirical data aligns with the simulation findings: GPS-based CTMM and camera-trap methods can yield similar estimates of DTD, whereas GPS-based SLD is strongly biased by temporal resolution and should not be relied upon for absolute movement metrics.

**Table 2:** Absolute and relative differences in estimated daily travel distance among movement estimation methods

Location	Species	Method comparison	Absolute difference (km/day)	Relative difference (%)
Bohemian Switzerland	Red Deer	CT vs. CTMM	0.1	1.8
		CT vs. SLD	4.1	76.4
		CTMM vs. SLD	4.0	74.9
Bavaria	Red Deer	CT vs. CTMM	-0.3	3.9
		CT vs. SLD	5.2	100.7
		CTMM vs. SLD	5.5	103.5
Bavaria	Wild Boar	CT vs. CTMM	-1.1	13.2
		CT vs. SLD	3.6	59.7
		CTMM vs. SLD	4.7	71.5

## Discussion

Two primary methods exist for estimating daily travel distance (DTD) in free-ranging animals: Lagrangian approach with biologging (e.g. GPS telemetry) and Eulerian approach with stationary sensors (e.g. camera traps). In this study, we directly compared DTD estimates from both approaches to assess whether they can provide consistent results. Using simulated, thus known movement trajectories, we tested how well each method recovered true DTD under the simulated movement and observation condition. We found strong agreement between camera-trap–based estimates and GPS-based estimates derived from continuous-time movement models (CTMM), while straight-line displacement (SLD) computed on the raw sequence of GPS locations consistently produced biased, scale-dependent under- and overestimates. The same comparisons were applied to empirical data collected, pairing the two techniques across three ungulate populations with spatio-temporally overlapping GPS and camera trapping datasets. We found that camera-trap–based and GPS-based CTMM estimates were of similar magnitude with overlapping confidence intervals, whereas GPS-based SLD produced lower values, and patterns that closely resembled those seen in the simulations. Together, these findings indicate that both methods under the conditions examined here, can yield accurate and similar estimates of daily travel distance (DTD) when appropriately analysed. Specifically, camera-trap data require correction for activity and detection bias, and the assumption that speed is consistent throughout the active period must hold or be corrected for [11]. Although camera-trap speed estimates were simulated without error in our study, there is currently no established method to quantify and propagate these errors into speed and DTD estimates, and this requires further evaluation. By contrast, GPS data analysis must, and can, account for positional error through appropriate error modelling.

In our simulations, GPS-based CTMM estimates remained largely scale-insensitive across subsampling intervals up to 60 min, consistent with earlier findings that CTMM performs reliably at moderate fix intervals [18]. At coarser resampling (≥ 120 min), accuracy declined. This decline is expected because coarse subsampling sharply reduces the number of short‐lag observations relative to the movement autocorrelation timescale, limiting the information available to identify velocity structure [21]. The effect was amplified by the finite track length of our simulations, however, extending simulated tracks to 70 days provided marginal improvement, indicating that a substantial increase in GPS duration would be required to off-set this bias (see Supplementary information S2; Fig. S2). Even at finer sampling intervals, improved precision and modest bias did not translate into a high confidence-interval coverage. This apparent discrepancy likely arose because the simulated trajectories were generated from a common process that captured movement features relevant to both estimators but did not exactly satisfy the assumptions of either. As a result, each estimator may converge on a pseudo-true value, θ*, that differs slightly from the generating value, θ. When precision increases, confidence intervals narrow around θ*, so even a small persistent bias can substantially reduce coverage of θ (Supplementary Information S2; Tables S1–S2). Thus, the low coverage appears to reflect confidence intervals narrowing around a pseudo-true value that differs slightly from the generating value, rather than poor performance of the point estimates, which remained relatively precise and only modestly biased at finer sampling intervals.

Although GPS-based CTMM estimates had narrow confidence intervals, they were based on only ten individuals (due to computational limitations). This was not problematic in our low-variability simulations, but in real populations with substantial inter‐individual heterogeneity such limited sample sizes can reduce precision and lead to unrepresentative population‐level estimates, especially if sex and age class structure of the population is not mirrored by the collared animals [41]. This may partly explain why wild boar in Bavaria showed the weakest agreement between the two methods, as the GPS-based analysis included only four collared individuals (Fig. 4; Table 2). However, with the available data, we cannot determine whether this discrepancy primarily reflects limited representation of population-level movement by the collared sample or a genuine difference between the two estimation methods in this system. More generally, larger and more representative samples of tagged animals are needed to distinguish these possibilities and support robust population-level inference, especially in systems with high movement variability.

By contrast, the GPS-based SLD estimates show the expected scale dependence, overestimation at short temporal fix intervals (due to measurement error; see [19]) and strong underestimation at coarse intervals (because path tortuosity is lost, [17]). SLD performance, however, depends strongly on the combination and magnitude of sampling interval, path tortuosity and positional error. SLD can yield reasonable estimates in specific conditions when movement is strongly directional and displacement between fixes is large relative to error [42]. These conditions are uncommon for most terrestrial mammals, and they also extend the range of fix intervals over which CTMM provides scale-insensitive estimates. Together, these results emphasize that CTMM provides reliable, scale-consistent estimates of DTD when fix interval and sample representation are adequate, effectively overcoming the scale-dependent biases inherent to SLD.

We found that camera-trap estimates of DTD can be precise but were influenced by both the number of sequences (a function of effort, abundance, and detection-related factors) and in particular, the accuracy of the diel activity estimation. Because detection probability increases approximately linearly with movement speed (except at very high speeds; [10]), periods of low movement were underrepresented. Combined with resting phases and assumptions of the movement model, this led to systematic underestimation of activity and, consequently, of DTD (Fig. 4c, see also Supplementary information S2; Fig. S1). In our simulations, this bias was amplified by the specific activity pattern used: a “flat-top” activity schedule with relatively abrupt transitions into and out of resting (speed forced to 0). Under this non-smooth data-generating process, the smooth circular kernel model used to estimate activity can overshoot near the transition windows and redistribute probability mass away from the plateau, further underestimating activity; this effect is smaller (or absent) when the underlying activity varies smoothly. In principle, bias from diel variation in speed can be reduced by modelling the time-of-day pattern in measured movement speed (and/or effective detection area) and using it as an inverse-weighting term when estimating activity [11], provided this relationship can be estimated reliably. Such a diel speed structure is plausible in empirical systems because movement metrics (e.g., velocity or step length) can vary with time of day [43–45]).

Estimating and correcting for diel variation in instantaneous speed can be difficult in practice because available data sources operate at different temporal scales and have biases. GPS data are often collected at relatively coarse sampling frequencies due to battery constraints — especially in medium-to-small sized species [46–49] — and, because they provide only discrete positions, effectively average movement over the intervals between fixes. This obscures diel patterns in instantaneous speed arising from within–fix-interval variation in movement. In principle, this limitation can be partly mitigated by dead-reckoning approaches that combine GPS with high-frequency accelerometer data (e.g. Dewhirst et al., [50]). Camera traps record movement at the near-instantaneous scale, but observed speeds are biased toward higher values because faster-moving animals are more likely to pass in front of a camera [11]. As a result, when speed and activity are positively correlated (i.e., animals move more slowly during low-activity periods), camera-trap speed samples can be sparse at low speeds and may not recover the true speed–time pattern, particularly when instantaneous speed is highly variable. However, several comparisons between camera-trap–derived and biologging-based activity curves [27, 51], together with the agreement of our empirical DTD estimates (Fig. 5), indicate that camera-trap–based estimates can perform well under field conditions without correction. Although true activity cannot be independently verified in such comparisons, the close correspondence across methods suggests that these estimates are generally robust despite potential sources of bias.

A separate source of uncertainty arises from the estimation of speed from camera-trap images, in our simulations, the speed of each camera-trap sequence was perfectly estimated, whereas in field practice, these measurements are subject to considerable error arising from calibration inaccuracies and user annotation variability (even though there are some qualitative checks on accuracy, see [26]). Together, these factors represent a fundamental — and likely underappreciated — source of bias or uncertainty in real-world camera-trap based DTD estimates warranting further investigation. In addition, camera-trap estimates may be biased if animals detect and respond to cameras (e.g., by changing speed), violating independence between observer and observed [52]. In principle, this bias can be reduced by discarding sequences (or segments within sequences) that show clear reactions, provided sufficient non-reactive observations remain for robust estimation; in our analyses we applied this filtering step, although it currently requires additional manual screening and therefore adds complexity to the processing pipeline that increasingly relies on AI-based recognition of species caught by cameras [53].

Taken together, these sources of uncertainty motivate two directions for future research: (i) testing when instantaneous speed conditional on being active is approximately time-invariant versus when it varies over the day, potentially through changes in the mix of gaits and behaviours, using camera traps paired with GPS dead-reckoning approaches based on high-frequency inertial sensors (e.g. Bidder et al., [54], Dewhirst, [50]); and (ii) improving the estimation and quantification of camera-trap–derived speed error through experimental camera-trap setups with known movement speeds that disentangle annotation error from calibration error, for example by using controlled experiments with known movement speeds and repeated independent calibrations and annotations.

Our results have direct implications for the Random Encounter Model (REM), which depends critically on reliable estimates of DTD. In REM, density is estimated as an inverse function of DTD [10], meaning that relative error in movement estimates translates directly into relative error in density estimates. Consequently, underestimating DTD by 10% inflates estimated density by approximately 10%, whereas overestimating DTD produces corresponding underestimation of density. A recent systematic review revealed that 91% of REM density estimates in predator-prey studies were based on DTD values that did not meet the model’s assumptions [55]. Quite often, detections came from non-randomly placed cameras (e.g., on trails) thus violating the independence of camera locations with respect to animals’ movement. Moreover, in many cases, DTD estimates were taken from unrelated populations, derived from sparse GPS data using SLD, or obtained from temporally mismatched data. Such practices are problematic because reliable REM estimates depend on DTD reflecting the same spatial and temporal context as the camera survey. For instance, Henrich et al., [24] showed that temporal (i.e. seasonal) mismatch between movement and observation data can bias DTD estimates, even when CTMM is applied. Our study demonstrates that, when spatial and temporal overlap is ensured and appropriate observation models are used, both camera trapping and CTMM can generate similar DTD estimates, thereby reducing a major source of bias in REM density estimation.

An important open question is whether and how camera trapping and GPS-based methods can be used together rather than separately to produce more accurate estimates. The two methods are inherently complementary: GPS telemetry often provides many data points to estimate the movement process of a small and potentially non-representative sample of individuals over extended time scales [41], whereas camera traps capture broader population-level variation but with greater uncertainty. Integrating these data streams has been identified as a key step forward in movement ecology and wildlife monitoring [56–62]. Our study shows that, at the scale of DTD and under the conditions examined here, camera-trapping- and GPS- based estimates can be directly comparable, suggesting they may share an underlying likelihood. This finding opens the door for joint modelling frameworks where both data sources contribute to the same estimation process. Hierarchical frameworks that combine individual-level GPS data with population-level camera detections (e.g. joint likelihood or data integration models; [61, 63]) particularly well suited for this task, as they allow the explicit specification of the observation models required to make the two data streams comparable. Integrating GPS telemetry analysed with CTMM and camera-trap data analysed using activity-corrected size-weighted log-normal models can exploit the complementary strengths of both approaches. Such synthesis would provide more robust, representative estimates of the movement process.

An important caveat of the empirical comparison is that the representativeness of the sampled individuals could not be formally evaluated. This limitation applies to both telemetry and camera-trap data: telemetry data necessarily came from a limited number of collared individuals, while camera-trap detections generally do not provide reliable individual-level demographic information. As a result, although both datasets were collected within the same spatial and temporal context, we cannot determine the extent to which they captured population-level variation in movement behaviour. This uncertainty is inherent to most empirical studies and contrasts with the synthetic case, where the composition and representativeness of the sampled population were known by design.

## Conclusion

In conclusion, our study provides the first systematic comparison of camera trapping- and GPS- based DTD under both simulated and real-world conditions with temporal and spatial overlap. We found that, when appropriately corrected and harmonized, both methods can yield similar estimates of DTD, whereas SLD remains unreliable due to its inherent scale dependence. These results highlight the potential for integration: GPS data offers precision, camera trapping provide population-wide coverage, and together they can deliver movement metrics that are both accurate and representative. As movement metrics are cornerstone parameters in various models of density, energetics, and habitat use, improving its estimation directly strengthens ecological inference and the reliability of management applications. A promising next step will be to extend these approaches to habitat-dependent variation in speed and DTD, where emerging frameworks that couple resource selection with velocity processes (e.g. Michelot and Hanks, [64]) may provide a natural route for integration. Beyond DTD, this may open opportunities to compare and integrate estimates of space use, occupation time, and animal responses to environmental features across GPS telemetry and camera-trap observation systems.

## Supplementary Information

Below is the link to the electronic supplementary material.

Supplementary Material 1

Supplementary Material 2

## Data Availability

The code used for the analyses and simulations is available on Zenodo ([Osinga, 2026] (https://doi.org/10.5281/ZENODO.21451022, https://anonymous.4open.science/r/Comparison-Daily-Travel-Distance-Estimation-FDB1/). Empirical GPS data from Bavaria National Park are available through the EURODEER and EUROBOAR repositories (https://euromammals.org/eureddeer/ and https://euromammals.org/euroboar/), accessible upon user registration. The camera trap data are included in the repository. Empirical data from Bohemian Switzerland are available in our repository.
