# Supplementary material for: Camera traps and GPS telemetry can provide similar estimates of daily travel distance in terrestrial mammals
Source: Mov Ecol. 2026 Jul 31;14:78. doi: 10.1186/s40462-026-00690-6 (PMC13732950; doi:10.1186/s40462-026-00690-6)
Supplement: Supplementary file 1 [file 40462_2026_690_MOESM1_ESM.docx]

**Journal:** Movement Ecology

**Title:** *Camera traps and GPS telemetry can provide comparable estimates of daily travel distance in terrestrial mammals*

**Authors:** *Thomas Osinga, Henrik J. de Knegt, Francesca Cagnacci, Maik Henrich , Marco Heurich, Astrid Olejarz, J. Marcus Rowcliffe, Massimo Scandura, Patrick A. Jansen*

**Supporting information S1**: Field methods for empirical data

**Czech Republic**

**Study area**

The study was conducted in the north-west part of the Czech Republic. Red deer equipped with GPS collars moved within the Bohemian Switzerland National Park area and east of the national park. Camera traps were placed east of the national park, overlapping with the space use of GPS collared red deer (50°50'6.690"N, 14°26'30.751"E; WGS 84). The landscape of the study area is shaped by sandstone rocks and the interconnected diversity of ecosystems (Varilova et al., 2011). The dominant forest type consists of coniferous stands, such as Norway spruce and Scots pine. The average annual air temperature lies between 6°C and 7°C (Varilova et al., 2011), fluctuating down to −20°C and up to 34°C. Mean annual precipitation in the study area remains relatively consistent, averaging close to 800 mm (Varilova et al., 2011). Ungulate populations are actively managed in the area (including through hunting/game management), and large carnivores occur (wolf and lynx).

**GPS telemetry**

The capture and handling of the red deer were approved by the Ethics Committee of the Ministry of the Environment of the Czech Republic, under number MZP/2019/630/361. The capture location corresponded to the supplementary feeding sites across the study area. Red deer was immobilised with the mixture of Ketamine and Xylazine using an airgun with an anaesthetic dart. A hybrid collar (Vectronic Aerospace GmbH) was mounted on the neck of the red deer, featuring GPS, a biologging sensor (Daily Diary tag from Wildbyte Technologies Ltd.), and a battery. The hybrid collar weighed 750g, lower than 3% of the red deer's body mass. The average time the hybrid collars remained on the red deer was up to two years. Fix frequency was set at 15 minutes. Fix intervals reduced when batteries ran low. When batteries were close to expiration, hybrid collars were either replaced or removed by an automatic drop-off system installed in the collars themselves.

**Camera trapping**

The camera trap (CT) survey was conducted between June 2024 and August 2024. We used trail CTs (Bushnell Model 119936C and Bushnell Model 119837) designated for unattended wildlife monitoring. Both CT models were equipped with infrared, motion-trigger and high-resolution photo capabilities. For the placement of the CTs, we first generated a 250 × 250 m grid covering the entire study area in the software QGIS. From this grid, we randomly selected 33 cells to serve as CT locations for animal detection. During the first sampling period, CTs were deployed in these randomly selected cells for a period of four weeks. After this first round, all CTs were moved one grid cell to the west and continued to operate for an additional four weeks, forming the second round of data collection. Secure digital memory cards were collected and replaced from the CTs after four and eight weeks. Pictures recorded by each CT were uploaded to the web-based platform Agouti for processing, annotating and archiving CT pictures. After a deployment calibration step, in the initial phase of picture annotation, the automatic model "Europe v5" was applied for species detection. The model has been developed by Agouti.eu, which is capable of detecting more than 100 species of mammals and birds that occur in Europe. In a second step, pictures, in which the AI model detected a wildlife species, were manually checked by an ecologist. If necessary, corrections were made based on sex, age, and group size. For each detected wildlife species with multiple observations, we created tracks of its movement, using an integrated function of Agouti, to perform the automatic estimation of animal day range. The CT setting was according to the standardised CT trapping protocol for wildlife density estimation created by the European Observatory of Wildlife (ENETWILD consortium et al. 2024).

**Germany**

**Study area**

The 230-km2 site of our camera trap study was located within the Bavarian Forest National Park in south-eastern Germany and also covered parts of the neighbouring Šumava National Park in the Czech Republic. The mountain range within the area encompasses an altitude between 570 and 1,453 m above sea level and is oriented from northwest to southeast. Annual mean temperatures decrease with elevation, from 7 to 3°C, and precipitation increases, from 1200 to 1850 mm (Ewald et al., 2011). The dominant habitat types are coniferous forest (43%) and mixed forest (31%) with Norway spruce (*Picea abies*), European beech (*Fagus sylvatica*) and white fir (*Abies alba*) (Cailleret et al., 2014; Pflugmacher et al., 2019). The study site within the national parks includes nature zones without forestry intervention (85% of the area), non-hunting zones (84%) and core zones within which human visitors are not allowed to leave the trails (49%). The red deer population is controlled by hunting between June and January, while roe deer have not been hunted since 2012. Natural predators of deer in the area are Eurasian lynx (*Lynx lynx*) and wolf (*Canis lupus*). This description is taken directly from Henrich et al., 2022.

**GPS telemetry**

**Wild boar**

Seven wild boar (1 adult male, 5 subadult males & 1 subadult female, 35 to 70 kg) were captured between February and August 2024 and equipped with a Vertex Plus GPS collar (Vectronic Aerospace, Berlin, Germany), which recorded a position every 30 min. Wild boar were captured at three different locations in the Bavarian Forest National Park, using wood-clad corral traps of ~30 m² equipped with live monitoring cameras and gates that close after the animals move a counterweight. We separated a caught wild boar in an attached metal cage and drove it into a net tunnel. Two or three people held it on the ground, with its eyes covered with a cloth (Linderoth et al., 2020). The entire procedure took ~5 min per animal, after which the wild boar was released at the capture site. An automated drop-off was activated after half year or a year depending on the size of the animal. The handling procedure was approved by the ethics committee of the Upper Bavaria government and met animal welfare requirements (permit number ROB-55.2-2532.Vet-02-20-149).

**Red deer**

Fifteen red deer (14 adult females & 1 adult male) were collared in the winter enclosure Riedlhäng on 20.01.2024 and	equipped with a Vertex GPS Plus collar (Vectronic Aerospace, Berlin, Germany), recording a position once per hour. One day every week fix frequency was set at 5 minutes. Fix intervals reduced when batteries ran low. Collars remained attached for a maximum of two years before automatically dropping off near the end of their battery lifespan. The deer were captured in catching corrals, allowing handling without immobilization. Animal handling and marking was approved by the Ethics Committees of the Government of Upper Bavaria (permit number: ROB-55.2-2532.Vet_02-21-132 ).

**Camera Trapping**

Across the study area, 60 camera traps (Cuddeback G) in total were placed in a systematic random design for a deployment time of 1 year (October 2023–September 2024). Camera trap locations were randomly selected from the centroids of a 1-km^2^ grid placed over the entire study area. The goal was to place camera traps in at least 50% of the centroids for the study area. The camera traps were placed as close as possible within a radius of 25 m around the preselected coordinates, while allowing for a free field of view of at least 8 m. If the camera trap could not be placed within this radius, for example, due to water bodies or steep terrain, the location was shifted to the centroid of another randomly selected unoccupied grid cell. The camera traps were enclosed within CuddeSafe boxes, which were fastened to tree trunks or wooden poles 50 cm above the ground and oriented northwards (10°). The vertical angle was adjusted using small pieces of wood as shims to ensure that the field of view was parallel to the ground. Camera trap settings included a ‘wide’ field for the pyroelectric sensor, image bursts of three photos per sensor trigger (0.04% of the image bursts were incomplete and 0.1% contained five photos due to wrong settings) and a minimal delay between consecutive image bursts (‘FAP’: fast as possible). An infrared flash with a ‘far’ range was used for night photography (Henrich et al., 2020). Every 24 h, a time lapse image was taken to check whether the camera traps worked continuously during a deployment or if there were interruptions. If a camera trap stopped working during a deployment, it was assumed that animal photos could have been taken until the last day with a time lapse image. The data processing followed the same ENETWILD workflow as the Czech Republic site.

**Map of study area with trajectories of animals used in the analysis.**

Figure S1**.** Study areas and data sources for the two empirical case studies. GPS fixes from individually tracked animals (points) and camera trap deployment locations (triangles) are shown for Czechia (left; Bohemian Switzerland, Czech Republic) and Bavaria (right; Bavarian Forest, Germany). In Bavaria, red deer (*Cervus elaphus*) and wild boar (*Sus scrofa*) were monitored concurrently across an overlapping camera trap array. Only individuals with sufficient data for continuous-time movement model fitting were retained for analysis (Czechia: *n* = 10 red deer; Bavaria: *n* = 14 red deer, *n* = 5 wild boar). Background maps show topographic relief to illustrate the landscape context of each study area.

**References**

Cailleret, M., Heurich, M., and Bugmann, H. (2014). Reduction in browsing intensity may not compensate climate change effects on tree species composition in the Bavarian Forest National Park. For. Ecol. Manag. 328, 179–192. doi: 10.1016/j.foreco.2014.05.030

ENETWILD-consortium, T Guerrasio, D Carniato, et al. 2024. “Generating Wildlife Density Data across Europe in the Framework of the European Observatory of Wildlife (EOW).” EFSA Supporting Publications 21 (10): 9084E. https://doi.org/10.2903/sp.efsa.2024.EN-9084.

Ewald, J., Jehl, H., Braun, L., and Lohberger, E. (2011). Die Vegetation des Nationalparks Bayerischer Wald als Ausdruck von Standort und Walddynamik. *Tuxenia* 31, 9–38.

Henrich, M., Hartig, F., Dormann, C. F., Kühl, H. S., Peters, W., Franke, F., Peterka, T., Šustr, P., & Heurich, M. (2022). Deer Behavior Affects Density Estimates With Camera Traps, but Is Outweighed by Spatial Variability. *Frontiers in Ecology and Evolution*, *10*. <https://doi.org/10.3389/fevo.2022.881502>

Linderoth, P., Johann, F., Handschuh, M., Bauch, T., Elliger, A., Dalüge, G., Herbst, C., Pegel, M., & Arnold, J. (2020). *Schwarzwildproblematik im Umfeld von Schutzgebieten. Raum-Zeit-Verhalten und Aktivität von Wildschweinen (Sus scrofa) in Gebieten mit Jagdruhezonen*. Projektbericht, Wildforschungsstelle Baden-Württemberg beim LAZBW.

Pflugmacher, D., Rabe, A., Peters, M., and Hostert, P. (2019). Mapping pan-European land cover using Landsat spectral-temporal metrics and the European LUCAS survey. *Remote Sens. Environ.* 221, 583–595. doi: 10.1016/j.rse.2018.12.001

Vařilová, Z., Navrátil, T. & Dobešová, I. (2011). *Recent Atmospheric Deposition and its Effects on Sandstone Cliffs in Bohemian Switzerland National Park,* Czech Republic. Water Air Soil Pollut 220, 117–130. https://doi.org/10.1007/s11270-010-0739-8
