# Supplementary material for: Camera traps and GPS telemetry can provide similar estimates of daily travel distance in terrestrial mammals
Source: Mov Ecol. 2026 Jul 31;14:78. doi: 10.1186/s40462-026-00690-6 (PMC13732950; doi:10.1186/s40462-026-00690-6)
Supplement: Supplementary file 2 [file 40462_2026_690_MOESM2_ESM.docx]

**Journal:** Movement Ecology

**Title:** *Camera traps and GPS telemetry can provide consistent estimates of daily travel distance in terrestrial mammals*

**Authors:** *Thomas Osinga, Henrik J. de Knegt, Francesca Cagnacci, Maik Henrich , Marco Heurich, Astrid Olejarz, J. Marcus Rowcliffe, Massimo Scandura, Patrick A. Jansen*

**Supplementary information S2**: Figures supporting the manuscript

Figure S1: Estimated activity from simulated camera-trap data as a function of the number of sequences used for activity estimation, shown under three amplitudes of circadian speed modulation: (a) amplitude = 0, (b) amplitude = 0.2 (used in main results), and (c) amplitude = 0.4. The red dashed line indicates the true activity, the blue line shows the mean across simulations (LOESS smoother), and grey points represent mean values from individual simulation runs.

Figure S2: Estimates of daily mean travel distance (DTD) from simulated telemetry data analysed using CTMM under different subsampling intervals (x-axis) across three different study lengths (panels). The redline indicates true DTD, the grey dots indicate individual runs and black dots are the mean between simulations. The whiskers indicate 95% percentile intervals.

Figure S3: Estimated daily travel distance (DTD) using GPS-based CTMM from simulated trajectories. The red dashed line marks the mean true DTD across simulations. “No correction” uses 5-min subsampled tracks with no measurement error correction; “Error-corrected” uses 5-min subsampled tracks with measurement error correction. Whiskers show 95% percentile intervals of the across-simulation mean.

Figure S4: Comparison of standard errors (SE) for daily travel distance (DTD) estimated using the delta method and a joint bootstrap procedure. Each point represents a unique simulation scenario differing in camera-trap sampling effort and activity estimation uncertainty. Point size indicates the number of speed sequences available for speed estimation. The dashed line denotes the 1:1 relationship, where delta-method and bootstrap SE estimates are identical. Across scenarios, delta-method SEs closely tracked bootstrap-derived SEs, with only modest deviations occurring under low-sample-size conditions. This agreement suggests that the delta method provides a reasonable approximation of uncertainty in DTD estimates while avoiding the substantial computational burden associated with joint bootstrap resampling.

Table S1: Summary of estimator performance across the simulation scenarios shown in **Figure 2**. The table reports the mean simulated daily travel distance (truth), mean estimated daily travel distance, bias, relative bias, root mean squared error (RMSE), precision (standard deviation of estimates), mean 95% confidence interval (CI) width, and coverage of the nominal 95% confidence intervals across simulation replicates for both scenarios representing the near-optimal sampling conditions.

| **Method** | **Mean estimate** | **population truth** | **Bias** | **Absolute bias** | **Relative bias (%)** | **Precision (SD)** | **RMSE** | **Coverage (%)** |
| --- | --- | --- | --- | --- | --- | --- | --- | --- |
| Camera Trap | 16.089 | 15.66 | 0.43 | 0.56 | 2.75 | 0.55 | 0.69 | 43.0 |
| GPS-based CTMM | 16.588 | 15.66 | 0.92 | 0.92 | 5.88 | 0.37 | 0.99 | 33.5 |

Table S2: Summary of estimator performance across the simulation scenarios shown in **Figure 3**. For each GPS sampling interval and estimation method (CTMM and SLD), the table reports the mean simulated daily travel distance (truth), mean estimated daily travel distance, bias, relative bias, root mean squared error (RMSE), precision (standard deviation of estimates), mean 95% confidence interval (CI) width, and coverage of the nominal 95% confidence intervals across simulation replicates.

| **Method** | **Interval (min)** | **Mean truth** | **Mean estimate** | **Bias** | **Relative bias (%)** | **RMSE** | **Precision (SD)** | **Mean CI width** | **Coverage (%)** |
| --- | --- | --- | --- | --- | --- | --- | --- | --- | --- |
| CTMM | 5 | 15.67 | 16.59 | 0.92 | 5.88 | 0.99 | 0.37 | 1.55 | 33.5 |
| CTMM | 15 | 15.67 | 16.58 | 0.92 | 5.86 | 0.96 | 0.31 | 1.31 | 23.5 |
| CTMM | 30 | 15.67 | 16.65 | 0.98 | 6.26 | 1.04 | 0.37 | 1.52 | 28.0 |
| CTMM | 60 | 15.67 | 16.87 | 1.20 | 7.68 | 1.34 | 0.59 | 2.49 | 50.0 |
| CTMM | 120 | 15.67 | 15.06 | -0.61 | -3.90 | 1.55 | 1.43 | 3.72 | 69.0 |
| CTMM | 240 | 15.67 | 10.68 | -4.99 | -31.84 | 5.12 | 1.16 | 3.26 | 4.0 |
| SLD | 1 | 15.67 | 26.35 | 10.69 | 68.22 | 10.69 | 0.29 | 1.09 | 0.0 |
| SLD | 2 | 15.67 | 19.02 | 3.36 | 21.42 | 3.37 | 0.31 | 1.29 | 0.0 |
| SLD | 3 | 15.67 | 16.62 | 0.95 | 6.06 | 1.00 | 0.31 | 1.36 | 21.0 |
| SLD | 4 | 15.67 | 15.27 | -0.40 | -2.53 | 0.51 | 0.32 | 1.40 | 80.0 |
| SLD | 5 | 15.67 | 14.57 | -1.10 | -6.99 | 1.13 | 0.30 | 1.26 | 7.0 |
| SLD | 15 | 15.67 | 10.91 | -4.76 | -30.37 | 4.77 | 0.30 | 1.25 | 0.0 |
| SLD | 30 | 15.67 | 8.74 | -6.93 | -44.23 | 6.93 | 0.27 | 1.16 | 0.0 |
| SLD | 60 | 15.67 | 6.73 | -8.94 | -57.06 | 8.94 | 0.24 | 1.00 | 0.0 |
| SLD | 120 | 15.67 | 5.04 | -10.62 | -67.82 | 10.63 | 0.19 | 0.81 | 0.0 |
| SLD | 240 | 15.67 | 3.66 | -12.01 | -76.66 | 12.01 | 0.15 | 0.64 | 0.0 |
